# Biochar-mediated uptake of secondary- and micro-nutrients in crops: a meta-analysis

**DOI:** 10.1039/d6ra03366k

**Published:** 2026-09-22

**Authors:** Manman Yuan, Jiabao Wang, Gang Wu, Pingping Wu, Chengshun Wang, Chuang Liu, Qi Miao, Yixiang Sun

**Affiliations:** a Key Laboratory of Nutrient Cycling and Arable Land Conservation of Anhui Province, Soil and Fertilizer Institute, Anhui Academy of Agricultural Sciences Hefei Anhui 230031 PR China mmyuan09@163.com sunyixiang@126.com

## Abstract

Secondary- and micro-nutrients are essential for the growth, high yield, and high-quality production of crops, and their deficiency in arable soils has become a global constraint in agriculture. As a novel soil amendment, biochar shows great potential in regulating soil nutrient availability and crop nutrient uptake, but its effects on crop secondary- and micro-nutrient acquisition are inconsistent due to varied biochar properties and environmental conditions. This meta-analysis systematically evaluated biochar effects on the uptake of seven key secondary- and micro-nutrients (Ca, Mg, S, Fe, Mn, Cu, and Zn) and the underlying regulatory mechanisms. Results showed that biochar significantly increased crop aboveground biomass and total secondary-/micro-nutrient accumulation without significantly altering their overall concentrations. Biochar exhibited element-specific effects: it reduced Mg, Mn and S concentrations, increased Fe, Cu, and Zn concentrations, and had no significant effect on Ca concentration. Biochar properties (pyrolysis temperature, feedstock, application rate, and pH) were the dominant regulators. The optimal conditions were 400 °C–500 °C pyrolysis temperature, wood-based feedstock, ≤1% (w/w) application rate, and alkaline pH, which affected the regulatory intensity but not the response direction. Soil properties, experimental methods, and crop types did not change the response pattern. Biochar stably regulated nutrient uptake across soils and crops, with the strongest promotion in vegetables. This study clarifies the universal regulatory characteristics of biochar and provides a scientific basis for its rational application to improve crop nutrient status in sustainable agriculture.

## Abstract 2

Biochar improves secondary and micronutrients accumulation in crops, especially vegetables.

## Graphical abstract

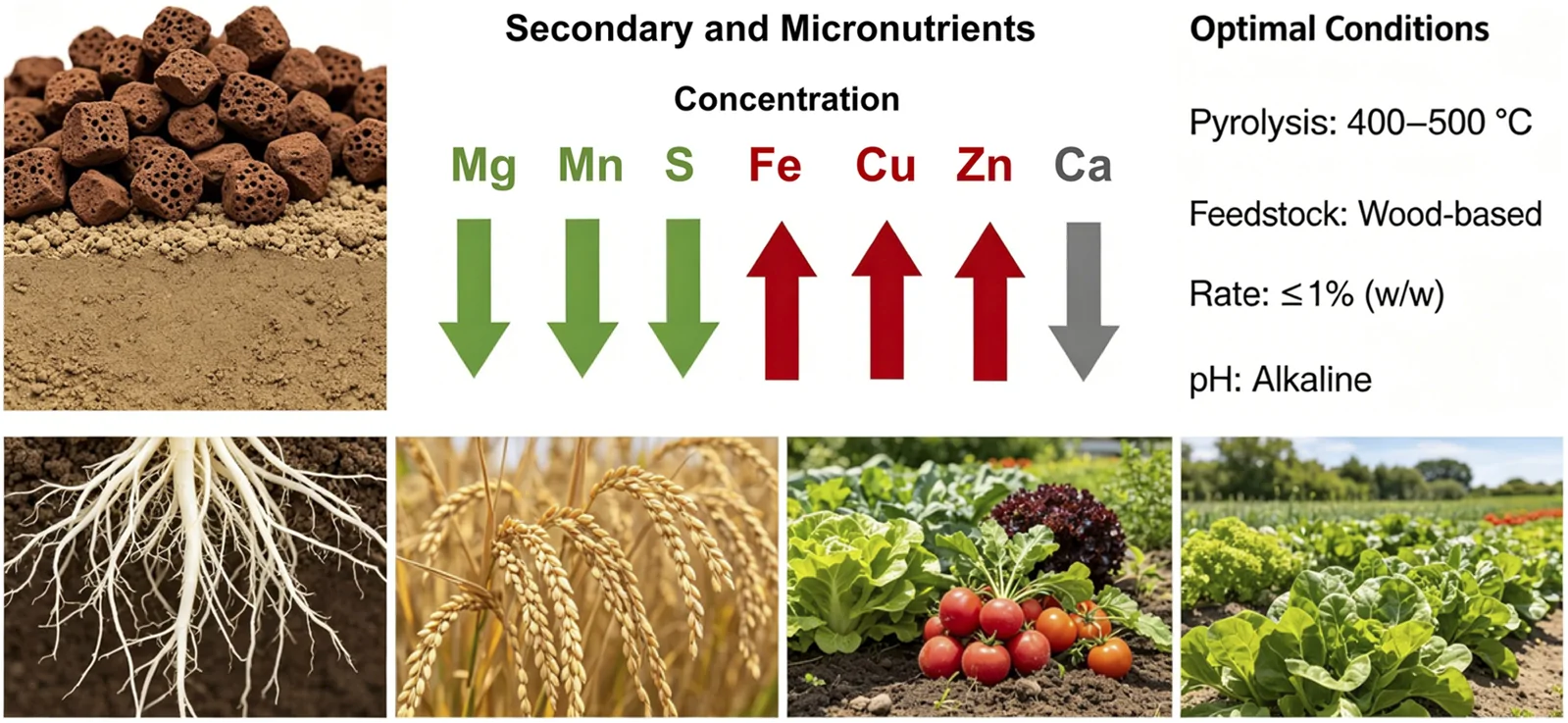

## 1 Introduction

Secondary- and micro-nutrients represent indispensable components for sustaining crop growth, elevating yield potential and enhancing product quality^1–4^ while serving as pivotal indicators for maintaining human dietary balance and physical health.^5^ Even though the concentrations of these nutrients in plant tissues are considerably lower than those of nitrogen, phosphorus and potassium, they participate extensively in the core physiological processes, including photosynthetic electron transport, enzymatic catalysis, substance metabolism, cell wall formation and antioxidant system establishment, thereby performing irreplaceable functions in crop development, stress tolerance and nutritional quality improvement.^6–13^ The insufficient supply of secondary- and micro-nutrients not only causes metabolic disorders, yield losses and quality degradation in crops but these deficiencies also propagate through the food chain to intensify the risks of malnutrition-related diseases in humans,^5^ highlighting the practical necessity of regulating the uptake of these nutrients by crops.

Crops mainly absorb secondary- and micro-nutrients from the soil, yet the deficient availability of these nutrients in global arable lands has evolved into a universal problem, restricting the high-quality and efficient development of modern agriculture. In agricultural production, the long-term excessive application of macronutrient fertilizers, accompanied by the negligence of secondary- and micro-nutrient supplementation, together with soil acidification, continuous cropping obstacles, nutrient leaching and immobilization, has further exacerbated the shortage of available secondary- and micro-nutrients in soils.^14,15^ Relevant studies indicate that approximately 40% of arable lands worldwide suffer from the deficiency of one or more secondary- and micro-nutrients, with this proportion exceeding 50% in developing countries.^16^ To alleviate this insufficient nutrient supply, the direct application of secondary- and micro-nutrient fertilizers is widely adopted in field production.^17^ Nevertheless, conventional fertilization practices suffer from problems such as low nutrient use efficiency, high vulnerability to loss and fixation, high input costs and potential risks of secondary soil pollution, making it difficult to achieve a precise, efficient and sustainable nutrient supply.^18^

Biochar is a functional soil amendment produced by biomass pyrolysis under oxygen-limited or oxygen-free conditions. With a well-developed pore structure, large specific surface area, abundant surface functional groups, strong cation exchange capacity and excellent nutrient adsorption–desorption performance, biochar presents broad application prospects in soil fertility improvement, nutrient regulation and crop yield enhancement.^19^ Numerous studies have confirmed that biochar can significantly affect nutrient uptake and accumulation in crops by improving soil physicochemical properties, increasing nutrient availability and promoting root growth and development.^20–24^ For secondary- and micro-nutrients, existing evidence indicates that biochar can modify the bioavailability of calcium (Ca), magnesium (Mg), sulfur (S), iron (Fe), manganese (Mn), copper (Cu), zinc (Zn) and other elements by adjusting soil pH, activating immobilized nutrients and reducing nutrient leaching loss, thereby imposing regulatory effects on their uptake by crops.^25–27^ However, current studies are mostly focused on individual elements, specific types of biochar or a narrow range of crop species and are mostly based on single-factor experiments. Consequently, research results are highly discrete with unclear regularities, and a systematic and unified understanding has not yet been formed.

The regulatory effects of biochar on the uptake of secondary- and micro-nutrients by crops are driven by both intrinsic properties and external environmental conditions. The feedstock type, pyrolysis temperature, application rate and pH of biochar directly determine its nutrient supply potential and soil amelioration intensity.^28,29^ In addition, soil properties such as pH and texture, as well as the nutrient demand characteristics of crops, significantly affect the magnitude and direction of regulatory effects.^21–24^ To date, a series of meta-analyses have systematically evaluated the effects of biochar on crop productivity, nitrogen uptake and utilization, and heavy metal immobilization in soils, laying a solid foundation for the rational agricultural application of biochar.^3,14,30^ As a statistical method for the quantitative integration of multiple independent studies, meta-analysis can effectively reduce the random errors and biases of single studies and improve the reliability and universality of conclusions.^31,32^ However, comprehensive, large-sample and multi-dimensional meta-analyses focusing on the overall regulatory effects of biochar on the uptake of multiple secondary- and micro-nutrients by crops, as well as the underlying mechanisms and universal patterns of key driving factors, are still insufficient. This gap limits the scientific guidance for applying biochar in the precise management of soil secondary- and micro-nutrients.

Accordingly, this study adopted meta-analysis to systematically synthesize published research data to (i) determine the overall effect of biochar on the concentrations and accumulation of seven key secondary- and micro-nutrients (Ca, Mg, S, Fe, Mn, Cu, and Zn) in crops; (ii) systematically analyze the regulatory patterns of biochar properties, soil conditions, experimental methods and crop types on nutrient uptake; and (iii) clarify the intrinsic mechanisms of biochar affecting the uptake of secondary- and micro-nutrients by crops.

## 2 Materials and methods

### 2.1 Data collection and compilation

Peer-reviewed articles published from January 2015 to December 2025 were systematically retrieved from major academic databases, including the Web of Science (https://www.webofscience.com), SpringerLink (https://link.springer.com), PubMed (https://pubmed.ncbi.nlm.nih.gov), Elsevier, Scopus and ScienceDirect (https://www.sciencedirect.com). A standardized search string was adopted to target relevant publications: “biochar” AND (“nutrient” OR “nutrition” OR “secondary nutrients” OR “micronutrients” OR “calcium” OR “Ca” OR “magnesium” OR “Mg” OR “sulfur” OR “S” OR “iron” OR “Fe” OR “manganese” OR “Mn” OR “copper” OR “Cu” OR “zinc” OR “Zn” OR “boron” OR “B” OR “molybdenum” OR “Mo”). Articles focusing on heavy-metal(loid) immobilization in contaminated soils, phytoremediation, or non-crop species were excluded to ensure data consistency with the present review scope. This meta-analysis only considered studies reporting biochar effects on the uptake of secondary- and micro-nutrients by crops in non-contaminated agricultural soils.

Following strict screening criteria, a final global dataset was established, comprising 144 paired observations from peer-reviewed field and pot experiments. The dataset covered 7 key secondary- and micro-nutrients: calcium (Ca), magnesium (Mg), sulfur (S), iron (Fe), manganese (Mn), copper (Cu), and zinc (Zn). To minimize publication bias, the treatment and control groups within each study were strictly paired under consistent experimental conditions (Table S1). The mean, standard deviation (SD), or standard error (SE) values were directly collected or calculated from the original literature. Data were extracted from the tables, figures, and supplementary materials of eligible publications. Quantitative data from graphs and figures were digitized using GetData Graph Digitizer 2.27.

To facilitate subgroup analysis and identify the driving factors regulating nutrient uptake, all observations were classified according to soil properties, biochar characteristics, experimental methods, and crop types following standard meta-analytic grouping protocols. Soil properties were classified according to texture and pH. Three soil texture groups were considered, namely, sand, loam and clay. Soil pH was divided into three categories: acidic, neutral, and alkaline. The biochar characteristics were assigned to feedstock types, pyrolysis temperature, application rate and biochar pH. Experimental methods were distinguished between pot and field experiments. Crop types were divided into three functional groups: cereals, vegetables, and other cash crops.

### 2.2 Meta-analysis

#### 2.2.1 Effect size calculation

The natural logarithm of the response ratio (ln RR) was adopted as the effect size to quantify crop responses to biochar amendment, calculated as follows:1
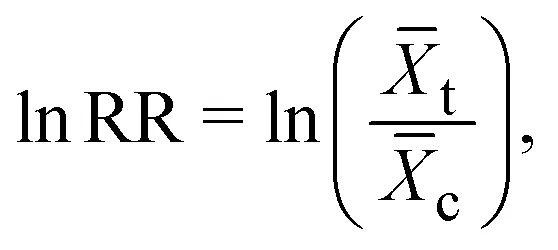
where *X̄*_t_ and *X̄*_c_ represent the mean values of the biochar treatment and control groups, respectively. The variance of ln RR was estimated following standard procedures for agricultural meta-analyses.^33,34^ The variance of ln RR was computed as follows:2
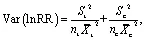
where *S*_t_, *S*_c_, *n*_t_ and *n*_c_ are the standard deviations and replicate numbers of the treatment and control groups, respectively.

#### 2.2.2 Overall effect and percentage change

Weighted mean effect sizes and 95% confidence intervals (95% CI) were generated using a random-effects model.^35^ The relative change (%) caused by biochar was calculated as follows:

3 Change in percentage (%) = (exp(ln RR) − 1) × 100

#### 2.2.3 Heterogeneity assessment

Heterogeneity was assessed using *Q* statistic and *I*^2^ statistic.^33,34^ High heterogeneity was defined as *I*^2^ > 50% and *P* < 0.05 for the *Q*-test, indicating that the responses were strongly modulated by the moderating factors.

## 3 Results

### 3.1 Overall effects of biochar on biomass and nutrient concentrations

To evaluate the overall effect of biochar across all experimental conditions (*i.e.*, pooling all observations regardless of biochar properties, soil types, experimental methods, and crop categories), we first examined the combined effect sizes (Table S2). The results showed no significant overall effects on aboveground biomass or the concentrations of Ca, Mg, Fe, Mn, and Zn (Table S2, *P* > 0.05), while the S concentration was significantly reduced (ln RR = −0.058, *P* < 0.01) and the Cu concentration was significantly increased (ln RR = 0.185, *P* < 0.05). Extremely high heterogeneity was observed across all variables (Table S2, *I*^2^ = 78.9–100%, *P* < 0.01), indicating that the overall pooled estimates mask the substantial variation driven by biochar characteristics, soil properties, experimental conditions, and crop types. Therefore, subgroup analyses were subsequently conducted to disentangle the effects of these modulating factors and reveal element-specific response patterns within each subgroup.

### 3.2 Effects of biochar on biomass and nutrient uptake among crop categories

#### 3.2.1 Biomass responses among crop categories

Biochar application increased aboveground biomass across all crop categories by 30.8% (Fig. 1, ln RR = 0.269, *P* < 0.01). Vegetables showed the highest increase by 42.1% (ln RR = 0.352), followed by other cash crops (33.2%; ln RR = 0.287) and cereals (24.4%; ln RR = 0.218).

**Fig. 1:**
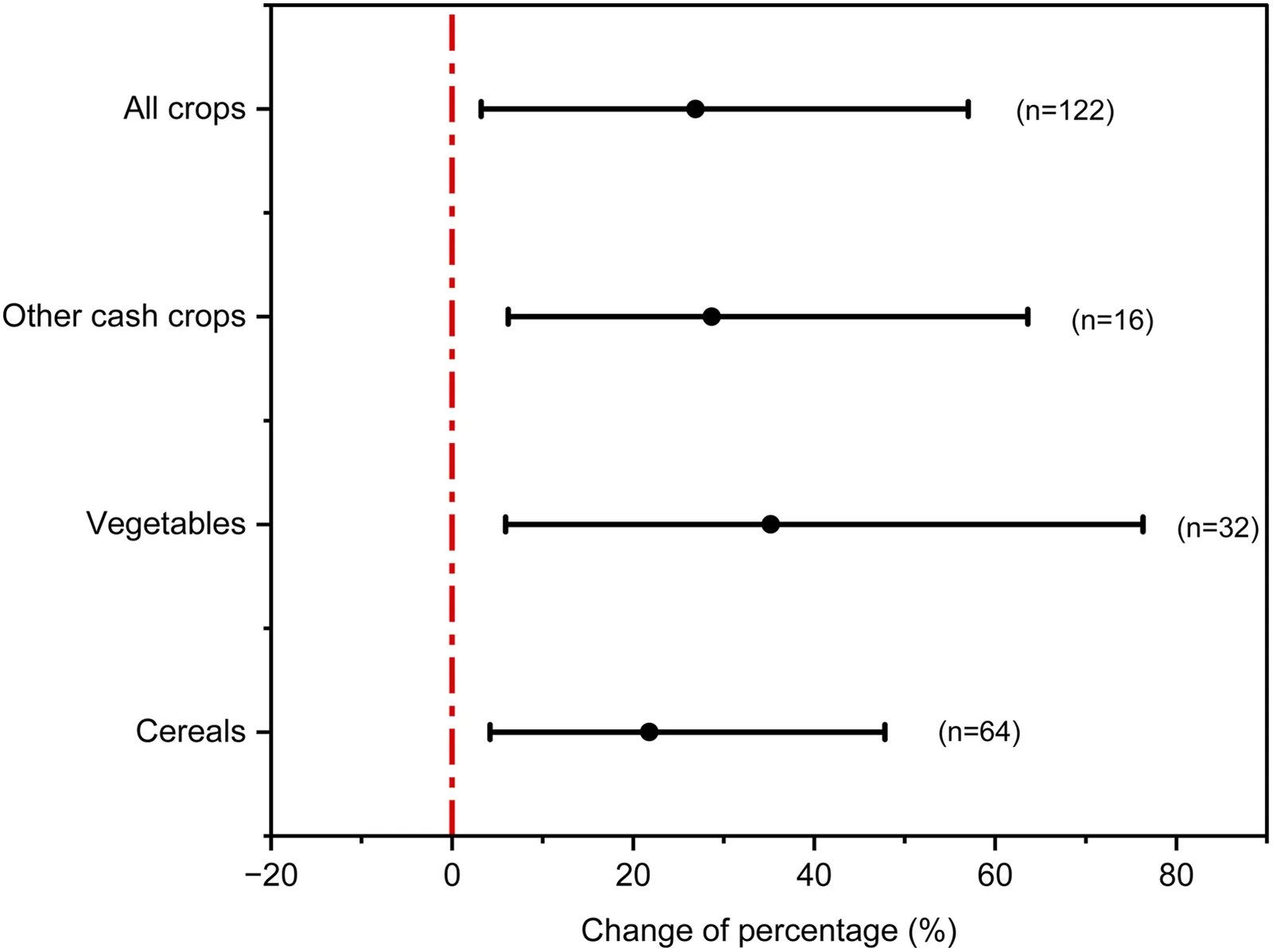
Effect of biochar application on crop biomass among different crop categories. Error bars represent 95% confidence intervals (95% CI), and *n* denotes the number of observations.

#### 3.2.2 Element concentration responses among crop categories

Across all crop categories, the total secondary- and micro-nutrient concentration was not significantly altered (Fig. 2a, ln RR = −0.006, *P* > 0.05). Biochar decreased Mg concentration by 8.3% (ln RR = −0.087, *P* < 0.05), Mn concentration by 15.5% (ln RR = −0.168, *P* < 0.01) and S concentration by 4.7% (ln RR = −0.058, *P* < 0.01) while increasing Fe concentration by 13.3% (ln RR = 0.125, *P* < 0.01), Cu concentration by 20.3% (ln RR = 0.185, *P* < 0.01), and Zn concentration by 9.6% (ln RR = 0.092, *P* < 0.01). No significant changes were observed for Ca concentration (Fig. 2a, *P* > 0.05).

**Fig. 2:**
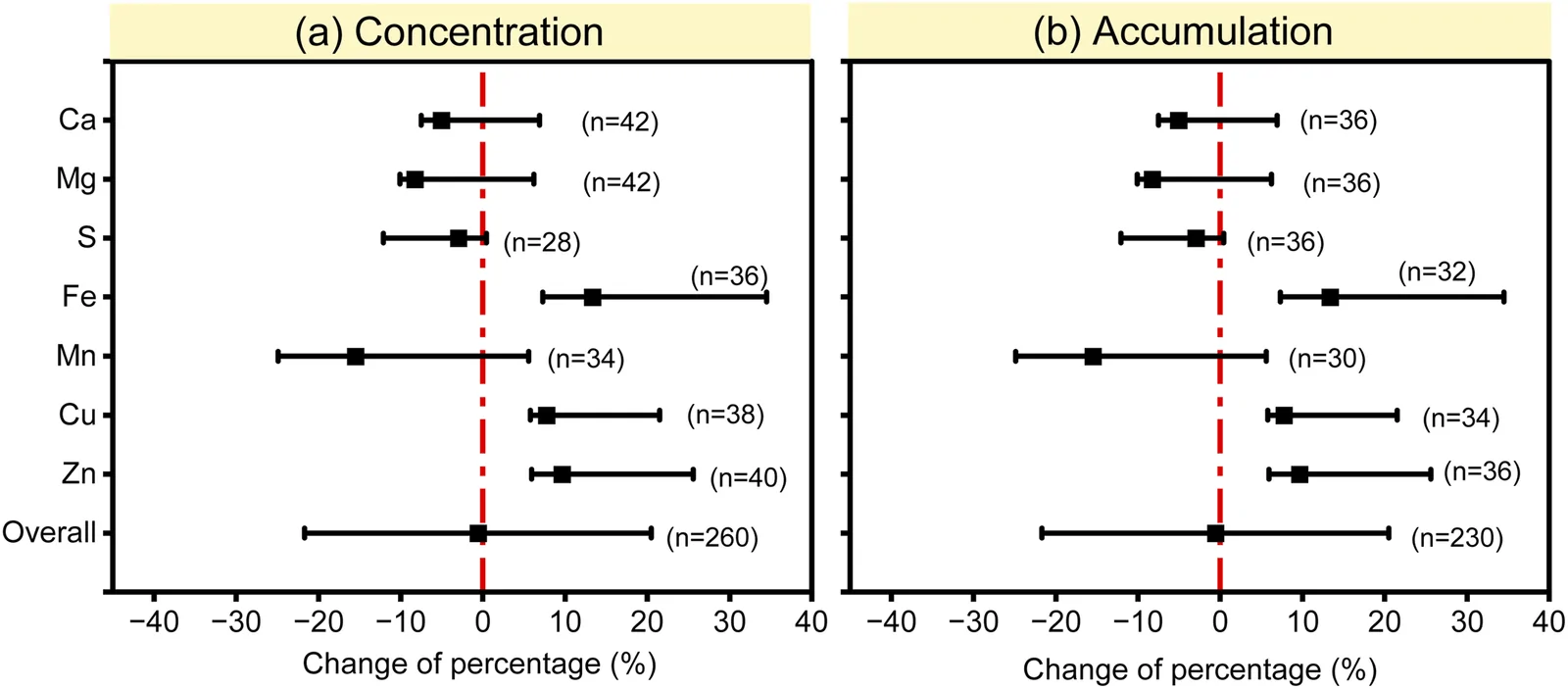
Effect of biochar application on element concentration (a) and accumulation (b) among different crop categories. Error bars represent 95% confidence intervals, and *n* denotes the number of observations.

#### 3.2.3 Element accumulation responses among crop categories

Biochar enhanced the total element accumulation by 12.8% (Fig. 2b, ln RR = 0.121, *P* < 0.01). The largest increases were found for Fe (23.9%), Zn (20.1%) and Cu (16.9%). Significant increases were also detected for S (11.4%), Mn (8.9%) and Ca (6.5%). Mg accumulation was not significantly altered (ln RR = 0.041, *P* > 0.05).

All crop categories showed consistent response patterns, with vegetables displaying the strongest responses for biomass and element accumulation.

### 3.3 Effects of biochar properties on nutrient concentration and accumulation

#### 3.3.1 Pyrolysis temperature

Biochar pyrolysis temperature of 400 °C–500 °C significantly increased the total element concentration (ln RR = 0.045, *P* < 0.05, Fig. 3a). Biochar pyrolysis temperature at ≤400 °C and >500 °C showed no significant overall effects on concentration (Fig. 3a, *P* > 0.05). All temperature regimes significantly decreased Mg and Mn concentrations and increased Fe, Cu, and Zn concentrations (Fig. 3a, *P* < 0.05). For all crop categories, the biochar pyrolysis temperature range significantly enhanced the accumulation of Ca, Mg, S, Fe, Mn, Cu and Zn, with the strongest effect at 400 °C–500 °C (Fig. 4a, ln RR = 0.125, 95% CI = 8.5–18.3%).

**Fig. 3:**
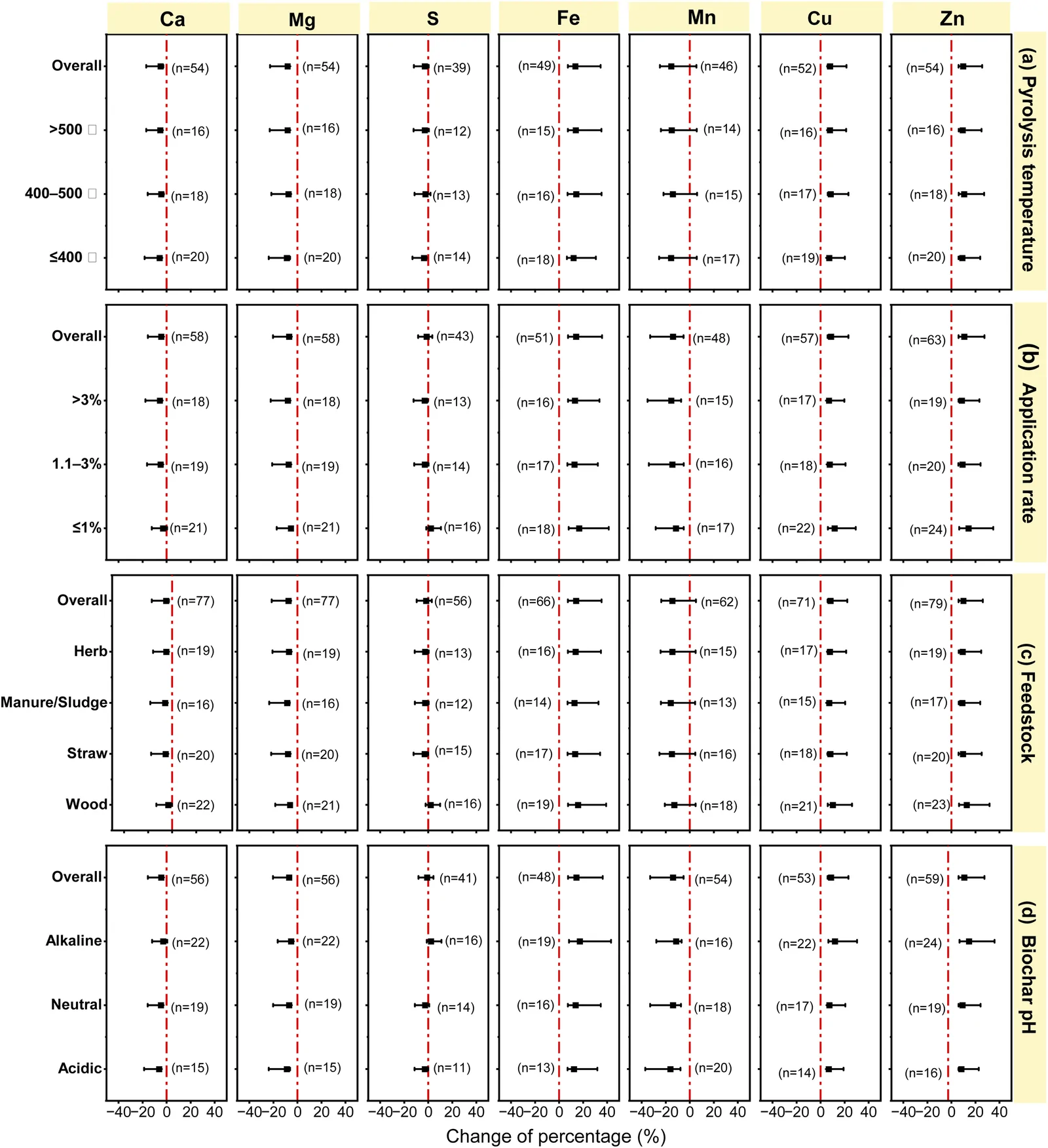
Effect of pyrolysis temperature (a), application rate (b), feedstock (c) and pH (d) of biochar on the concentrations of Ca, Mg, S, Fe, Mn, Cu and Zn in crops. Error bars represent 95% confidence intervals, and *n* denotes the number of observations.

**Fig. 4:**
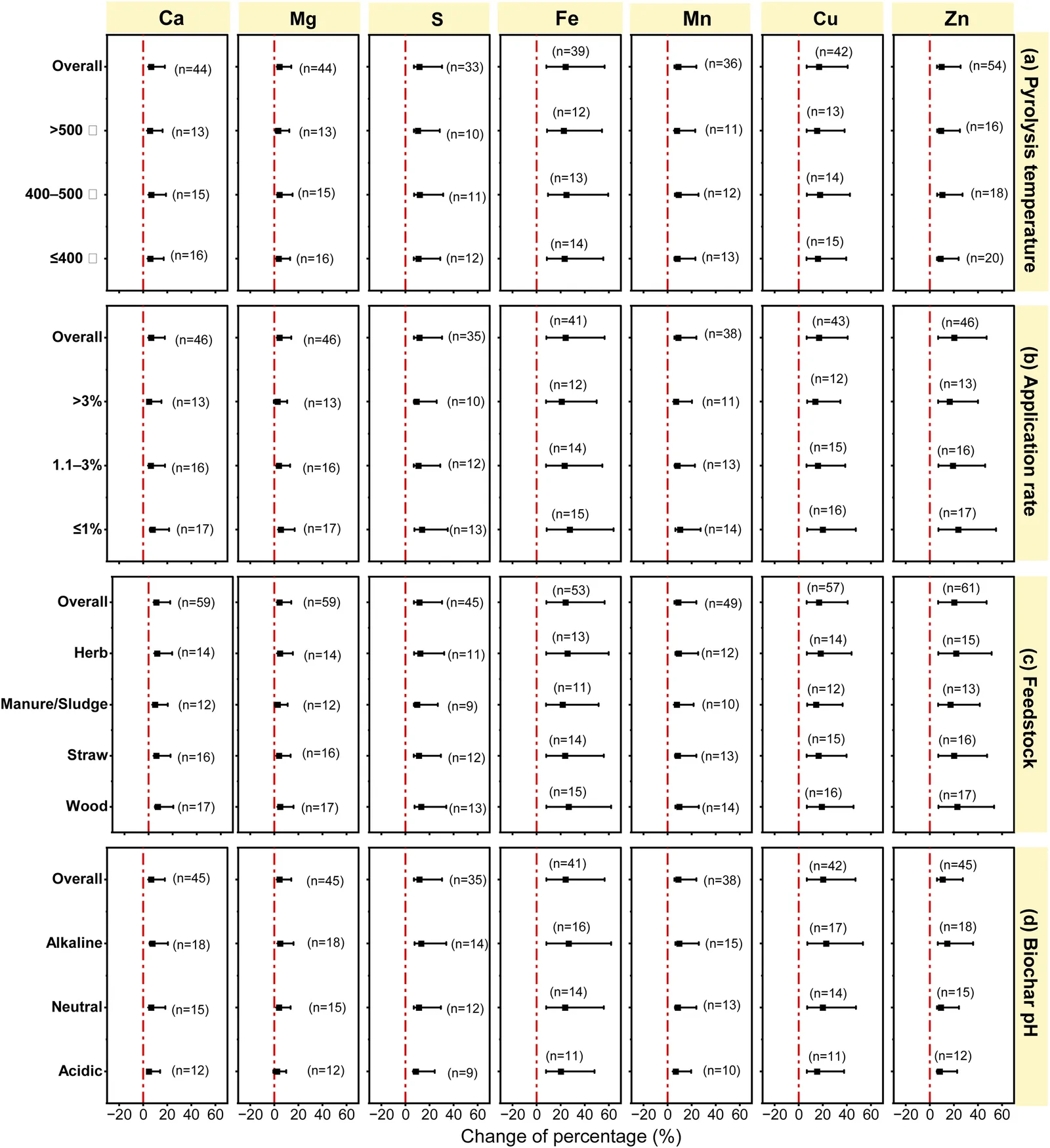
Effect of pyrolysis temperature (a), application rate (b), feedstock (c) and pH (d) of biochar on the accumulation of Ca, Mg, S, Fe, Mn, Cu and Zn in crops. Error bars represent 95% confidence intervals, and *n* denotes the number of observations.

#### 3.3.2 Feedstock type

Wood-based biochar significantly increased the concentrations of the seven secondary- and micro-nutrients (ln RR = 0.051, 95% CI = 1.1–9.5%, *P* < 0.05) and showed stronger effects on Fe, Cu, and Zn concentrations than straw-based, manure-/sludge-based, or herb-based biochars (Fig. 3c). All feedstocks significantly reduced Mg and Mn concentrations and improved the accumulation of the seven secondary- and micro-nutrients (*P* < 0.01), with wood-based biochar yielding the highest accumulation response (Fig. 4c, ln RR = 0.132, 95% CI = 9.2–19.2%).

#### 3.3.3 Application rate

A biochar application rate of ≤1% (w/w) produced the largest increase in the concentrations of the seven secondary- and micro-nutrients (Fig. 3b, ln RR = 0.055, 95% CI = 1.5–9.9%, *P* < 0.01) and their accumulations (Fig. 4b, ln RR = 0.138, 95% CI = 10.1–19.8%, *P* < 0.01). The effects on the concentration and accumulation of the seven secondary- and micro-nutrients gradually declined with increasing biochar application rate (Fig. 3b and 4b). All biochar application rates significantly decreased Mg and Mn concentrations and increased Fe, Cu, and Zn concentrations (Fig. 3b, *P* < 0.01).

#### 3.3.4 Biochar pH

Alkaline biochar significantly increased the concentrations of the seven secondary- and micro-nutrients (Fig. 3d, ln RR = 0.058, 95% CI = 1.8–10.4%, *P* < 0.01) and their accumulations (Fig. 4d, ln RR = 0.135, 95% CI = 9.8–19.4%, *P* < 0.01), with a weaker reduction in Mn concentration and accumulation than that induced by acidic and neutral biochars and stronger promotion of Fe, Cu, and Zn concentration and accumulation than that induced by acidic and neutral biochars (Fig. 3d and 4d, *P* < 0.05). All biochar pH categories significantly improved the accumulation of the seven secondary- and micro-nutrients (Fig. 4d, *P* < 0.01).

### 3.4 Effect of soil properties on nutrient uptake

#### 3.4.1 Soil pH

Soil pH did not alter the overall response direction of element concentrations. In acidic, neutral and alkaline soils, biochar consistently decreased Mg and Mn concentrations and increased Fe, Cu, and Zn concentrations (Fig. 5b, *P* < 0.05). Biochar increased the accumulation of the seven secondary- and micro-nutrients in acidic soils by 12.5% (ln RR = 0.118, *P* < 0.01), neutral soils by 13.0% (ln RR = 0.122, *P* < 0.01) and alkaline soils by 13.3% (Fig. 6b, ln RR = 0.125, *P* < 0.01).

**Fig. 5:**
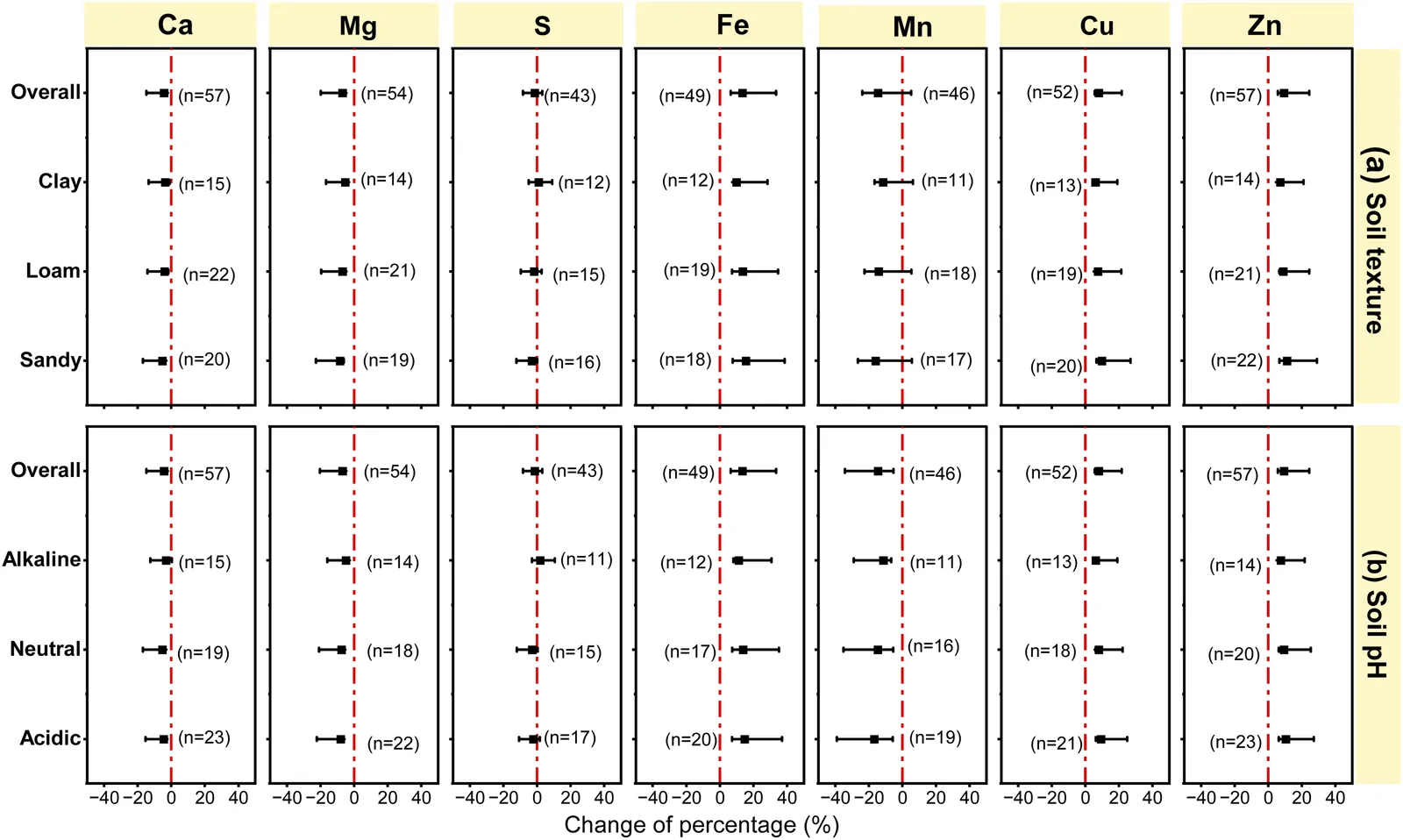
Effect of soil texture (a) and soil pH (b) on Ca, Mg, S, Fe, Mn, Cu and Zn concentrations in crops. Error bars represent 95% confidence intervals, and *n* denotes the number of observations.

**Fig. 6:**
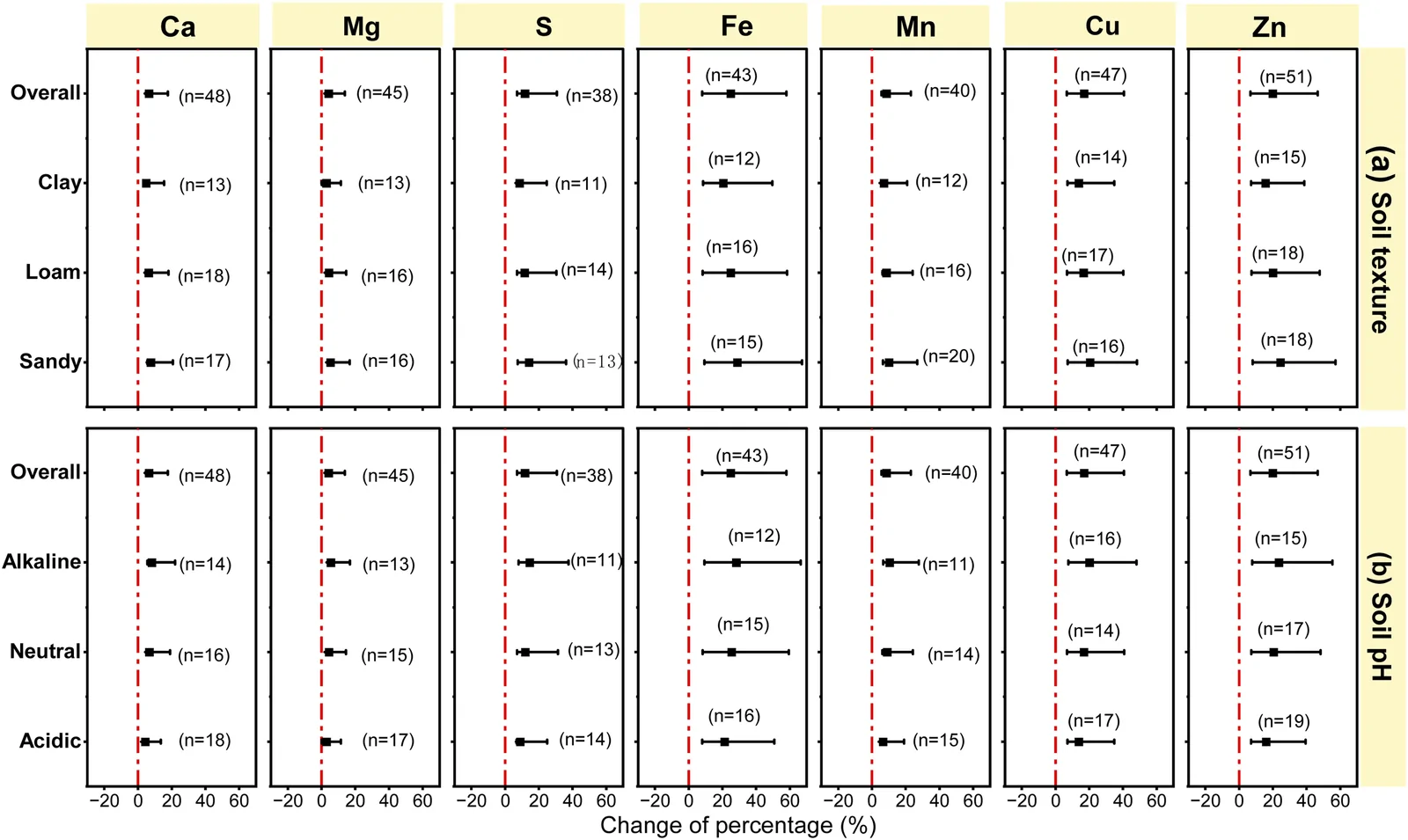
Effect of soil texture (a) and soil pH (b) on Ca, Mg, S, Fe, Mn, Cu and Zn accumulation in crops. Error bars represent 95% confidence intervals, and *n* denotes the number of observations.

#### 3.4.2 Soil texture

Soil texture (sandy, loam, and clay) did not significantly modify concentration responses to biochar. Biochar consistently reduced Mg and Mn concentrations and increased Fe, Cu, and Zn concentrations across all textures (Fig. 5a, *P* < 0.05). The accumulation of the seven secondary- and micro-nutrients increased significantly in sandy soil by 12.2% (ln RR = 0.115, *P* < 0.01), loam soil by 12.9% (ln RR = 0.121, *P* < 0.01) and clay soil by 13.1% (ln RR = 0.123, *P* < 0.01), with no significant differences among the textural classes (Fig. 6a).

### 3.5 Effects of experimental methods on nutrient uptake

#### 3.5.1 Pot *vs.* field experiments

Both pot and field experiments showed identical response directions: decreased Mg and Mn concentrations, increased Fe, Cu and Zn concentrations, and an unchanged total element concentration (Fig. 7a). Biochar significantly increased the accumulation of the seven secondary- and micro-nutrients in both experimental methods: pot by 13.1% (ln RR = 0.123, *P* < 0.01) and field by 12.6% (Fig. 8a, ln RR = 0.119, *P* < 0.01).

**Fig. 7:**
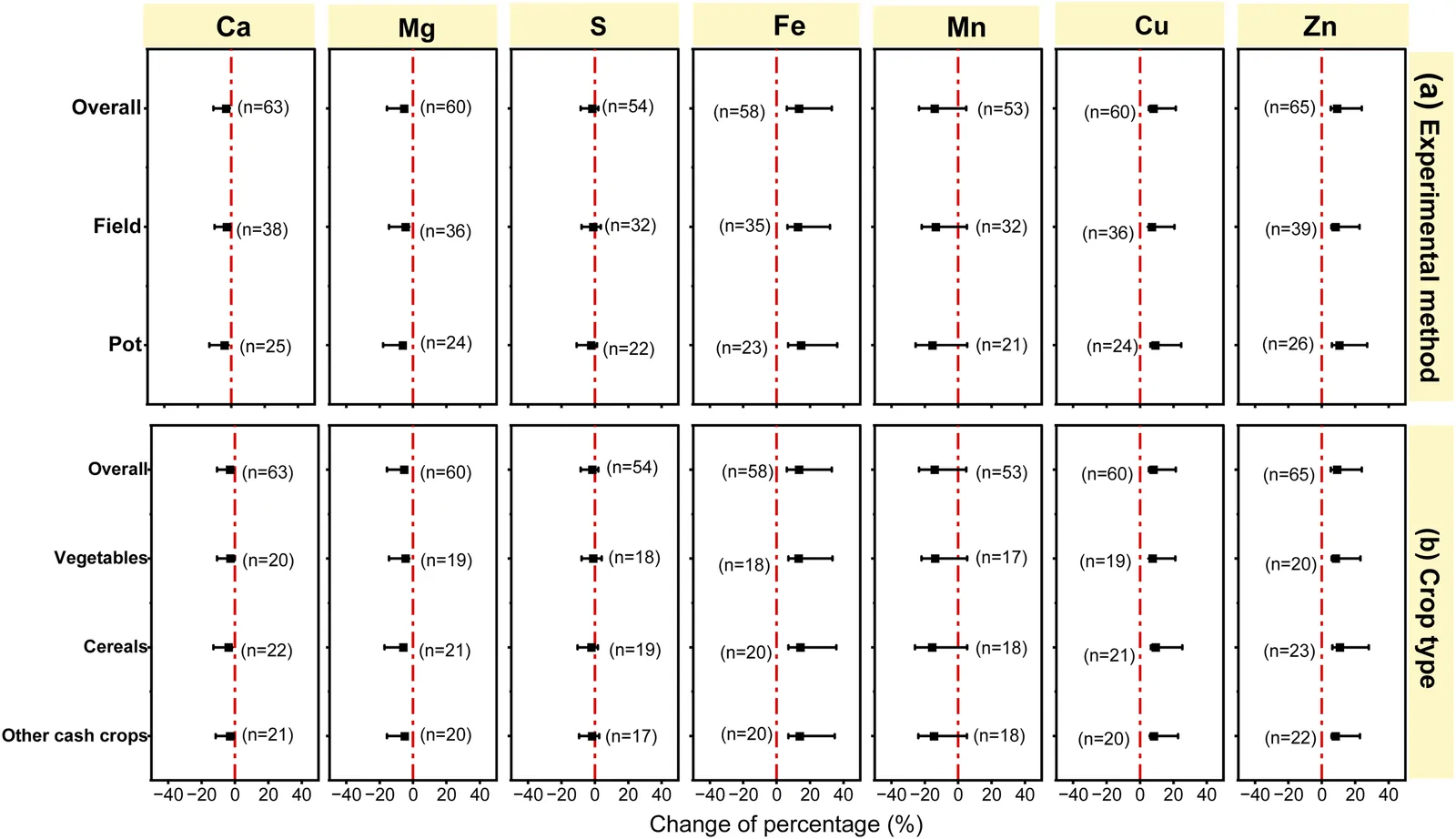
Effect of experimental method (a) and crop type (b) on Ca, Mg, S, Fe, Mn, Cu and Zn concentrations in crops. Error bars represent 95% confidence intervals, and *n* denotes the number of observations.

**Fig. 8:**
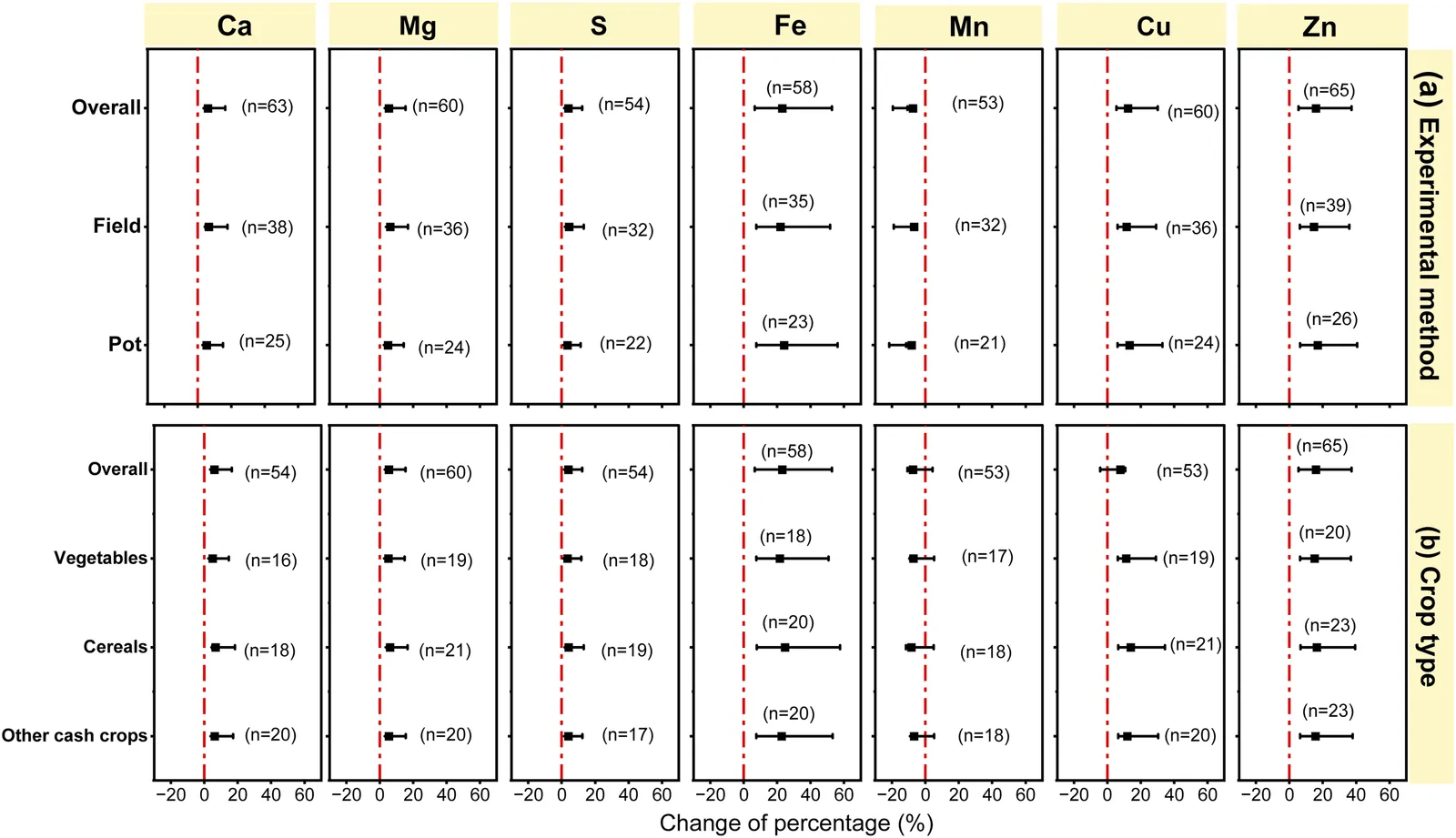
Effects of experimental method (a) and crop type (b) on Ca, Mg, S, Fe, Mn, Cu and Zn accumulation in crops. Error bars represent 95% confidence intervals, and *n* denotes the number of observations.

#### 3.5.2 Crop groups

To further verify the robustness of the crop-category response patterns observed in Section 3.2, we examined the interactions between the crop groups and experimental methods (Fig. 7b and 8b). The results confirmed that cereals, vegetables, and other cash crops exhibited identical response directions across both pot and field experiments: biochar consistently decreased Mg and Mn concentrations while increasing Fe, Cu, and Zn concentrations (Fig. 7b, *P* < 0.05). Vegetables consistently exhibited stronger responses than cereals and other cash crops across both experimental methods.

## 4 Discussion

### 4.1 Overall effects of biochar on crop biomass and nutrient uptake

The application of biochar significantly increased crop aboveground biomass by an average of 30.8% across all included observations, with the most pronounced enhancement observed in vegetables, followed by other cash crops and cereals. This stimulation of biomass production represents the primary mechanism underlying the significant increase in the total accumulation of secondary- and micro-nutrients (12.8%), while their overall concentrations remained largely unchanged. Mechanistically, biochar improves soil organic matter content and cation exchange capacity, enhances soil water and nutrient retention capacity, and provides a stable nutrient supply for crop growth.^26,36^ Moreover, biochar optimizes soil pH, alleviates root growth inhibition caused by extreme soil acidity or alkalinity, promotes root elongation and branching, and expands the root nutrient-absorbing area, thereby strengthening the nutrient acquisition capacity of crops.^37^ In addition, biochar itself contains inherent nutrients that can be directly utilized by crops, collectively contributing to the increase in crop biomass.^22,38^ The enhanced biomass generation leads to a “dilution effect” on nutrient concentrations,^39^ yet the total nutrient uptake by crops is significantly elevated, thus presenting the characteristic of “stable concentration but increased accumulation”.^37,38^

At the individual element level, biochar exerted distinct element-specific regulation: it significantly decreased Mg, Mn and S concentrations, significantly increased Fe, Cu and Zn concentrations, and exerted no significant effects on Ca concentration. Such element-specific responses are predominantly determined by the chemical speciation of nutrients in the soil and the selective adsorption–desorption behaviors of biochar toward different elements.^40^ In soil, Mg and Mn mainly exist in exchangeable forms;^41^ the elevation of soil pH following biochar application readily induces the precipitation of Mg and Mn into their insoluble hydroxides or carbonates, thereby reducing their bioavailability and consequently inhibiting crop uptake.^4,22^ In contrast, Fe, Cu and Zn are prone to being fixed in soil in the forms of oxides or hydroxides; the abundant functional groups on biochar can form stable complexes with Fe, Cu and Zn, alleviate their immobilization, improve their bioavailability, and simultaneously promote root growth, thus significantly enhancing the uptake of these three micronutrients by crops.^22,38^ Due to its high mobility and relatively stable bioavailability in soil, Ca is minimally affected by biochar amendment, resulting in no significant change in its concentration.^25,26^

From a plant physiological perspective, the element-specific regulation by biochar further involves root uptake and within-plant translocation processes. Ca, present mainly as exchangeable Ca^2+^, is absorbed *via* non-selective cation channels and translocated to shoots *via* the xylem, driven by transpiration.^6^ Its high mobility accounts for the limited regulatory effect of biochar on Ca concentration. Mg is taken up through the MRS2/MGT family transporters,^42^ and biochar-induced pH elevation promotes Mg precipitation, thereby reducing its availability. S, predominantly present as sulfate (SO_4_^2−^), enters the roots *via* the high-affinity SULTR transporters and is translocated as sulfate or glutathione.^43^ Biochar can reduce plant-available S by enhancing sulfate leaching and microbial immobilization. Fe, largely fixed as Fe(iii) oxides in soil, is acquired through strategy I (reduction-IRT1 pathway) or strategy II (phytosiderophore chelation).^10^ Biochar surface functional groups can complex Fe(iii) and promote its reductive dissolution. Mn is absorbed *via* the NRAMP family transporters.^11^ Elevated pH favors Mn oxidation into insoluble forms. Cu is taken up through the COPT/CTR transporters and heavy-metal ATPases (HMAs).^1^ The labile Cu complexes formed by biochar increase the phytoavailable Cu pool. Zn is absorbed *via* the ZIP family transporters.^42^ Biochar mobilizes fixed Zn through ligand-promoted dissolution. These physiological processes, in conjunction with biochar-induced changes in the soil chemical environment, collectively determine the element-specific responses observed.

### 4.2 Regulation effects of biochar properties on nutrient uptake

#### 4.2.1 Pyrolysis temperature

Pyrolysis temperature exhibited a clear threshold effect on nutrient uptake, with 400 °C–500 °C identified as the optimal range. Biochar produced at this medium-temperature range develops a well-defined porous structure and possesses abundant surface functional groups (*e.g.*, hydroxyl and carboxyl), which substantially enhance the adsorption and retention of soil-available nutrients, reduce nutrient leaching loss, and promote the dissolution of insoluble nutrient fractions, thereby increasing soil-available nutrient concentrations.^44^ Additionally, medium-temperature biochar features a moderate carbonization degree and high inherent nutrient content, which can directly supply diverse secondary- and micro-nutrients for crop absorption. By comparison, low-temperature biochar (≤400 °C) suffers from incomplete carbonization and exhibits an underdeveloped pore structure, scarce functional groups and a weak nutrient adsorption capacity, resulting in a limited promoting effect on nutrient uptake.^37^ High-temperature biochar (>500 °C) undergoes severe pore structure collapse and loss of surface functional groups, and its strong alkalinity tends to induce the immobilization of partial nutrients, reducing nutrient bioavailability.^37^ Notably, all pyrolysis temperature regimes consistently decreased Mg and Mn concentrations and increased Fe, Cu and Zn concentrations,^26^ confirming that the element-specific differential regulation is a stable and universal feature of biochar independent of pyrolysis temperature.

From a thermochemical perspective, the mineral nutrients retained in biochar undergo distinct transformations during pyrolysis at 400 °C–500 °C. At this temperature range, most alkali and alkaline earth metals (Ca and Mg) and micronutrients (Fe, Mn, Cu, and Zn) remain thermally stable as their oxide and carbonate decomposition temperatures generally exceed 500 °C.^22^ However, their chemical speciation shifts markedly. Specifically, the organically bound and water-soluble fractions in the raw feedstock are converted into oxide, carbonate, or silicate forms embedded in the biochar matrix.^13,22^ The moderate pyrolysis temperature thus strikes a balance. It is sufficiently high to convert nutrients into stable, slow-release mineral forms that persist in the soil yet not so high to cause severe volatilization or sintering that would reduce nutrient bioavailability.

#### 4.2.2 Feedstock type

Wood-based biochar displayed the strongest regulatory efficacy on secondary- and micro-nutrient uptake, significantly increasing the concentrations and accumulation of the seven target nutrients, with a more pronounced promoting effect on Fe, Cu and Zn than that induced by straw-based, manure-/sludge-based and herb-based biochars. The underlying reason lies in the differences in the inherent nutrient content, pore structure and impurity level among biochars derived from different feedstocks.^26^ Wood-based biochar forms a well-developed pore structure and possesses abundant functional groups after pyrolysis, which effectively adsorb and retain soil-available nutrients.^45^ Straw-based and herb-based biochars possess a moderate nutrient content and adsorption capacity, leading to relatively weak regulatory effects. Manure-/sludge-based biochar contains not only certain secondary- and micro-nutrients but also high levels of heavy metal impurities and inert substances that compete with crops for nutrient uptake, coupled with a poorly developed pore structure, resulting in the weakest regulatory effect.^13,26^ All feedstock types significantly enhanced the accumulation of the seven nutrients and exhibited identical element-specific regulation patterns, indicating that the feedstock type only affects the magnitude of biochar's regulatory effect without changing the response direction of nutrient uptake.

#### 4.2.3 Application rate

Biochar application rate showed a clear dose effect on nutrient uptake, with ≤1% (w/w) determined as the optimal application rate that significantly increased the concentration and accumulation of the seven nutrients; the promoting effects gradually declined with the increase in the application rate.^46^ A low application rate (≤1%) moderately improves soil physicochemical properties and accelerates the dissolution of insoluble soil nutrients, while a high rate (>3%) excessively elevates soil pH and intensifies nutrient immobilization. Meanwhile, a low rate balances soil nutrient supply, avoids nutrient antagonism, and facilitates the synergistic uptake of various secondary- and micro-nutrients by crops.^21^ In contrast, a high application rate (>3%) excessively elevates soil pH, causing the precipitation of Ca, Mg and other cations into their insoluble forms and reducing their bioavailability.^47^ It also significantly increases soil organic matter content, intensifying the complexation of Zn with organic matter and decreasing the soil CaCl_2_-extractable Zn content.^48^ Furthermore, a high rate enhances the adsorption and immobilization of certain nutrients by biochar, thereby inhibiting crop nutrient uptake.^37^ In some cases, it may even cause soil compaction and impair root respiration and growth.^37^ Consequently, the promoting effect of biochar on nutrient uptake is significantly weakened. All application rates consistently decreased Mg and Mn concentrations and increased Fe, Cu and Zn concentrations, further verifying the universal element-specific regulation of biochar.^22^

#### 4.2.4 Biochar pH

Alkaline biochar presented the strongest promoting effect on nutrient uptake, significantly increasing the concentrations and accumulation of the seven nutrients. It exerted a weaker reducing effect on Mn concentration and accumulation and a stronger promoting effect on Fe, Cu and Zn concentrations and accumulation than acidic and neutral biochars. Alkaline biochar optimizes soil pH, particularly in acidic soils, neutralizes soil acidity and mitigates the leaching loss of Ca, Mg and other elements induced by low pH.^38^ Meanwhile, it reduces the precipitation of Fe, Cu and Zn into insoluble oxides or hydroxides, thus significantly improving their bioavailability.^49^ Alkaline biochar is also rich in surface functional groups that form stable complexes with secondary- and micro-nutrients, thereby not only reducing nutrient leaching but also facilitating the root uptake and translocation of these nutrients.^50^ Acidic biochar has a limited capacity to adjust soil pH and cannot effectively enhance the bioavailability of soil secondary- and micro-nutrients, coupled with fewer surface functional groups, leading to a weak promotional effect on nutrient uptake.^51^ Neutral biochar exerts a moderate effect on soil pH adjustment and nutrient adsorption, with its regulatory capacity falling between acidic and alkaline biochars.^52^ All pH types of biochar significantly improved the accumulation of the seven elements,^22,23^ indicating that biochar can reliably promote crop biomass increase and thus elevate total nutrient accumulation regardless of its intrinsic pH.

The long-term persistence of biochar in soil and the nutrient content inherent to its feedstock are two interrelated factors that modulate its regulatory effects on secondary- and micro-nutrient availability. Biochar is dominated by aromatic carbon structures that are highly resistant to microbial decomposition, enabling it to persist in the soil for extended periods and maintain residual improvements in soil fertility, including nutrient retention and pH buffering.^3,21^ Meta-analytic evidence has shown that biochar amendment can sustainably increase the soil-available phosphorus, potassium, and total nutrient contents over multiple growing seasons.^3^ With respect to feedstock origin, the inherent nutrient content of the biomass source directly determines the nutrient-enrichment potential of the resulting biochar.^3,21^ During pyrolysis, organic matter is volatilized, while mineral elements (including Ca, Mg, Fe, Mn, Cu, and Zn) are concentrated in the ash fraction, leading to nutrient enrichment relative to the raw feedstock.^22^ Biochars derived from manure or sludge generally contain higher levels of these mineral nutrients than wood- or straw-based biochars, although they may also carry higher heavy metal loads.^13^ Thus, the choice of feedstock not only shapes biochar's physical and chemical properties but also determines its inherent capacity to supply secondary- and micro-nutrients to crops over long term.

### 4.3 Regulation of soil properties by biochar-mediated nutrient uptake

#### 4.3.1 Soil pH

In acidic, neutral and alkaline soils, biochar consistently decreased Mg and Mn concentrations and increased Fe, Cu and Zn concentrations while significantly enhancing the accumulation of the seven nutrients. The slight difference in the promoting magnitude of nutrient accumulation is related to the initial bioavailability of soil nutrients. In acidic soils, low pH triggers severe leaching of Ca, Mg and S, and Fe, Mn, Cu and Zn are easily fixed as oxides, leading to the low bioavailability of most secondary- and micro-nutrients.^44^ Biochar (especially alkaline biochar) adjusts soil pH to an optimal range, dissolves insoluble nutrient fractions, adsorbs and retains available nutrients, and reduces leaching loss, thus significantly boosting crop nutrient uptake.^38^ Neutral soils have a relatively sufficient initial bioavailability of secondary- and micro-nutrients. Biochar mainly improves soil structure and promotes root growth, resulting in a moderate promotional effect on nutrient accumulation.^21,22^ In alkaline soils, high pH causes Ca, Mg, Mn and other elements to precipitate as their insoluble forms. Biochar cannot effectively modify the high-pH soil environment. Root nutrient uptake and translocation are impaired under alkaline conditions.^53^ Even so, biochar can still adsorb and retain partially available nutrients and promote root growth, exerting a significant promoting effect on nutrient accumulation. More broadly, biochar amendment has been demonstrated to improve soil physicochemical properties, including pH, cation exchange capacity, organic carbon content, and available nutrient content, and enhance secondary- and micro-nutrient acquisition in various cropping systems,^21^ underscoring the universal role of soil-property modification in biochar-mediated nutrient regulation.

#### 4.3.2 Soil texture

Soil texture (sand, loam, or clay) did not significantly modify the response patterns of nutrient concentrations to biochar application. Biochar consistently reduced Mg and Mn concentrations and increased Fe, Cu and Zn concentrations across all soil textures and significantly enhanced the accumulation of the seven elements with no significant differences among textural classes. The regulatory mechanisms of biochar vary with soil texture, which is closely associated with soil water and nutrient retention capacity. Sandy soils have high porosity but poor retention, making secondary- and micro-nutrients prone to leaching. The porous structure of biochar greatly improves the water and nutrient holding capacity of sandy soils, adsorbs and retains available nutrients to reduce leaching,^26,54^ optimizes the soil structure and stimulates root growth, thus strongly promoting nutrient uptake. Loam soils possess moderate water and nutrient retention with stable bioavailable nutrient levels. Biochar exerts a relatively mild improvement in soil properties, leading to a moderate promotional effect on nutrient accumulation.^21,22^ Clay soils are characterized by low porosity and strong nutrient retention. Secondary- and micro-nutrients are easily immobilized by soil colloids. Biochar can adsorb and activate some of these immobilized nutrients, improve soil aeration and promote root growth, resulting in a significant promotion of nutrient accumulation.^36^

### 4.4 Regulatory effects of experimental methods and crop types on nutrient uptake

#### 4.4.1 Experimental methods

Pot and field experiments showed completely consistent response patterns of nutrient concentrations and accumulation to biochar application. Biochar decreased Mg and Mn concentrations, increased Fe, Cu and Zn concentrations, did not significantly change the total nutrient concentration, and significantly elevated the total nutrient accumulation. Pot experiments are conducted under controlled environments that are free from interference by natural factors such as rainfall and wind. Biochar is thoroughly mixed with soil, leading to a more pronounced regulatory effect on soil properties.^26^ In contrast, field experiments are affected by natural factors. Biochar is prone to migration and leaching under field conditions, and its mixing with soil is less uniform, slightly weakening its regulatory effect.^37^ The consistent results from pot and field experiments fully validate the reliability and reproducibility of biochar's regulatory role in secondary- and micro-nutrient uptake, providing a robust experimental foundation for the field application of biochar.

#### 4.4.2 Crop types

Cereals, vegetables and other cash crops exhibited identical response patterns to biochar. All three crop categories reduced Mg and Mn concentrations, increased Fe, Cu and Zn concentrations, and significantly elevated the total secondary- and micro-nutrient accumulation. Vegetables showed the strongest positive responses, followed by cereals and other cash crops.^22^ Such crop-specific differences are attributed to the variations in growth characteristics and nutrient uptake capacity among crop types.^48,55^ Most vegetables are fast-growing with short growth cycles and rapid biomass accumulation, leading to high overall demand and uptake rates for secondary- and micro-nutrients.^8,56^ They also develop root systems with dense root hairs, forming a large nutrient absorption area and exhibiting strong nutrient acquisition capacity, which make the promoting effect of biochar on their growth and nutrient uptake the most pronounced.^57,58^ Cereal crops have longer growth cycles and moderate nutrient demands. Their fibrous root systems result in a relatively weak nutrient uptake capacity,^59^ leading to a moderate response to biochar application. Other cash crops have distinct nutrient demands. Legumes such as soybean benefit from rhizobial nitrogen fixation, which optimizes the root microenvironment and enhances nutrient uptake.^60–62^ Cotton has high requirements for Ca and Mg, and thus, biochar exerts a relatively strong promotional effect on the absorption of these two elements.^49,56^ However, its overall growth rate and nutrient demand are lower than those of vegetables, and hence, its response magnitude is between that of cereals and vegetables.

### 4.5 Fundamental differences between biochar regulation of secondary-/micro-nutrients and heavy metals

Biochar differs fundamentally in regulatory targets and mechanisms between essential secondary- and micro-nutrients and toxic heavy metals (*e.g.*, Cd, Pb, and Cr). Secondary- and micro-nutrients are commonly deficient in farmland soils. Biochar can enhance the uptake and accumulation of these nutrients in crops by improving soil nutrient availability, optimizing soil structure, activating the immobilized nutrients, and promoting root growth, which in turn alleviate nutrient deficiency and improve the nutritional status of crops.^9,18,38,63^ For toxic heavy metals, the core goal of biochar is to reduce crop uptake by immobilizing heavy metals in soil through irreversible adsorption, precipitation and complexation, thereby lowering their bioavailable content and reducing heavy metal accumulation in edible parts to improve food safety.^40^

Although both processes depend on the porous structure and surface functional groups of biochar, their adsorption behaviors are essentially different.^42^ Biochar adsorbs secondary- and micro-nutrients in a reversible manner. It retains nutrients to prevent leaching and releases them slowly, supporting a sustained nutrient supply for crops. You *et al.*^45^ confirmed this characteristic in coastal soil, where wood chip biochar reversibly adsorbed soil Zn, Mn and B, thereby increasing their available content for a long time. In contrast, biochar adsorbs heavy metals irreversibly, firmly fixing metals in soil and preventing their re-release and uptake by plants.^64^ This essential difference requires differentiated biochar application strategies in agricultural production. For the regulation of secondary- and micro-nutrients, medium-temperature, wood-based, alkaline biochar at a low application rate is preferred, especially in acidic soils.^38,46^ For heavy metal remediation, high-temperature and high-porosity biochar is more suitable to maximize immobilization efficiency.

## 5 Limitations and prospects

### 5.1 Limitations

Despite rigorous literature screening and standard meta-analysis to ensure reliability, this study presents several limitations. First, the number of included studies remains limited, with a primary focus on cereals and common vegetables, while data for fruit trees and other cash crops are insufficient. Several studies only involve single elements, leading to unbalanced sample distribution, potential bias in effect-size estimation, and restricted universality of conclusions. Second, the included studies differ widely in pyrolysis temperature, feedstock type, application rate, and other experimental conditions. Although heterogeneity and sensitivity analyses were conducted, residual heterogeneity may still lead to minor deviations in the effect sizes of certain elements. Third, incomplete raw data in some publications required indirect calculation, which may reduce the accuracy of the quantitative results. Finally, this study only evaluates the single effects of unmodified biochar on seven secondary- and micro-nutrients. Synergistic or antagonistic interactions between biochar and other nutrient amendments and differences in crop nutrient uptake across various growth stages are not explored. Biochar modifications such as trace-element loading or acidification are also not included in the analysis, further constraining the scope and applicability of this research.

### 5.2 Prospects

To address the limitations and follow the trends in soil nutrient regulation, future research should focus on the following aspects. (i) Expand the scope of literature inclusion, give priority to understudied crops including fruit trees, balance sample distribution across crop types, and improve the comprehensiveness and universality of research conclusions. (ii) Explore the combined application of biochar with magnesium fertilizers, zinc fertilizers and other soil amendments, clarify the synergistic mechanisms underlying nutrient uptake, and incorporate differences among crop growth stages to support the precise field application of biochar. (iii) Conduct in-depth research on modified biochar materials and screen for optimal preparation formulations to further strengthen the promoting effects on the uptake of secondary- and micro-nutrients by crops. (iv) Integrate molecular biological techniques to investigate the regulatory impacts of biochar on the expression levels of nutrient transporter genes, revealing core regulatory mechanisms at the molecular level and enriching the theoretical framework.

## 6 Conclusion

This study systematically demonstrated the regulatory effects of biochar on the uptake and accumulation of Ca, Mg, S, Fe, Mn, Cu and Zn by crops. Biochar application significantly increased crop aboveground biomass, thereby elevating total secondary- and micro-nutrient accumulation without altering their overall concentrations. Biochar showed element-specific regulation by decreasing Mg, Mn and S concentrations, increasing Fe, Cu and Zn concentrations, and exerting no significant effects on Ca concentration. Biochar properties were the dominant influencing factors, and the optimal conditions were 400 °C–500 °C pyrolysis temperature, wood-based feedstock, application rate of ≤1% (w/w) and alkaline pH, which only affected the regulatory intensity rather than the response direction. Soil properties, experimental methods and crop types did not change the response pattern of nutrient concentrations to biochar application. Biochar stably regulated nutrient uptake across various soils and crops, with the strongest effect on vegetables, and showed high homogeneity in regulating S and Cu concentrations. These findings reveal the universal regulatory traits of biochar, and provide a theoretical basis and practical guidance for rational biochar application to improve soil nutrient availability and crop nutrient acquisition in sustainable agricultural production.

## Author contributions

Manman Yuan: conceptualization, methodology, data curation, writing – original draft preparation, writing – review and editing. Jiabao Wang: software. Wu Gang: validation. Chengshun Wang: data curation. Pingping Wu: visualization. Chuang Liu: supervision. Qi Miao: writing – review and editing. Yixiang Sun: conceptualization and funding acquisition.

## Conflicts of interest

There are no conflicts of interest to declare.

## Supplementary Material

RA-016-D6RA03366K-s001

RA-016-D6RA03366K-s002

## Data Availability

The data used in this meta-analysis were obtained from previously published studies which are fully cited in the reference list. The processed dataset and meta-analysis results are available within the manuscript and its supplementary information (SI). Supplementary information: Table S1: biochar-mediated uptake of secondary- and micro-nutrients in crops: a meta-analysis supplementary data; Table S2: heterogeneity and overall effect size (ln RR). ln RR, log response ratio; *k*, number of studies; *Q*, the *Q* statistic tests for heterogeneity among effect sizes; df, degrees of freedom for the *Q*-test; *P* < 0.05 (*), *P* < 0.01 (**), ns, not significant; and 95% CI, 95% confidence interval. See DOI: https://doi.org/10.1039/d6ra03366k.
