# Supplementary material for: Biochar-mediated uptake of secondary- and micro-nutrients in crops: a meta-analysis
Source: RSC Adv. 2026 Sep 22;16(44):56181–94. doi: 10.1039/d6ra03366k (PMC13595112; doi:10.1039/d6ra03366k)
Supplement: Supplementary file 1 [file RA-016-D6RA03366K-s002.pdf]

Supplementary Information (SI) for RSC Advances.
This journal is © The Royal Society of Chemistry 2026

Table S2. Heterogeneity and overall effect size (ln*RR*). ln*RR*, log response ratio; *k*, number of studies; *Q*, the *Q* statistic tests for heterogeneity among effect sizes; *df,* degrees of freedom for the *Q-test;* $P < 0.05$ (*), $P < 0.01$ (**), ns, not significant; 95% CI, 95% confidence interval.

**Variable**	***k***	***Q***	***df***	***P***	**I^2**	**Heterogeneity**	**Random ln*RR***	**95% CI**	**Significance**
Aboveground Biomass	113	4320.74	112	< 0.01	99.8%	Very high	0.353	[-15.9%, 86.4%]	ns
Ca concentration	75	2082.18	74	< 0.01	99.8%	Very high	0.127	[-20.4%, 45.9%]	ns
Mg concentration	73	2137.95	72	< 0.01	99.8%	Very high	0.302	[-32.5%, 92.8%]	ns
S concentration	14	359.08	13	<0.05	96.4%	Very high	-0.058	[−7.8%, −3.7%]	**
Fe concentration	48	75.01	47	< 0.01	96.0%	Very high	0.106	[-45.3%, 66.6%]	ns
Mn concentration	37	120.42	36	< 0.01	96.7%	Very high	0.117	[-19.4%, 42.8%]	ns
Cu concentration	51	326.74	50	< 0.01	78.9%	Very high	0.182	[13.1%, 28.0%]	*
Zn concentration	96	201.58	95	< 0.01	97.5%	Very high	0.079	[-15.6%, 31.4%]	ns
